# miR-181 suppresses metastasis via MMP-14

**DOI:** 10.18632/aging.100824

**Published:** 2015-10-20

**Authors:** Eric Roth, Jian Cao

**Affiliations:** Department of Medicine, Stony Brook University, Stony Brook, NY 11794, USA

**Keywords:** cancer, metastasis, matrix metalloproteinase, miRNA

Metastasis accounts for 90% of all cancer-related deaths. Much effort has been made to identify key molecules controlling metastasis. However, due to the complicated wiring of the cancer signaling networks, key players initiating cancer metastasis have not been defined. This situation has thwarted target-based drug discovery aimed at preventing cancer metastasis. Regardless of which molecules initiate tumor, cancer cells must be equipped with proteases for invasive behavior [1]. A key cell surface-anchored protease, membrane type 1-matrix metalloproteinase (MMP-14), plays a critical role in digesting basement membrane and extracellular matrices (ECMs) and in inducing cancer cell migration, thereby promoting cancer invasion and metastasis [2]. MMP-14 is highly expressed in most human cancers and correlates with breast cancer mortality [3]. However, the regulatory mechanism of upregulated MMP-14 expression in cancer is poorly understood. We, for the first time, demonstrated that MMP-14 is directly inhibited by miR-181a-5p through targeting the 3′ untranslated region (UTR) resulting in decreased cell migration, invasion, and angiogenesis [4]. This relation introduces the possibility of miR-181a-5p as a biomarker for prognosis, or as a target to prevent cancer metastasis.

MicroRNAs (miRNA or miR) are a relatively new element in gene regulation that have been shown to regulate several aspects of metastasis. They are short noncoding RNAs which are transcribed as approximately 70 nucleotide precursors, and are processed to a 22 nucleotide mature form. It is the mature form that enacts genetic regulation, primarily through post transcriptional repression. Although some studies suggest miRNAs enhance expression by binding to the promoter region, most exert their effect by binding the 3′ UTR of transcribed mRNA, thereby signaling degradation or preventing protein translation. This regulatory element has been shown to play an important role in the transformation to malignancy [5].

Using two miRNA target prediction algorithms, TargetScan and miRanda, a putative miR-181a-5p binding site within the 3′-UTR of MMP-14 was identified. Overexpression of miR-181a-5p reduced ectopically expressed 3′UTR containing-MMP-14 mRNA and protein; however, it had no effect on MMP-14 lacking the 3′ UTR. In order to decrease endogenous miR-181a-5p levels, a sponge construct with four repeats containing miR-181 binding sites was generated. When stably integrated, the sponge decreased highly expressed endogenous miR-181a-5p in MCF-7 cells, resulting in increased expression of hypoxia-induced endogenous MMP-14. These effects of overexpression and knockdown both suggest miR-181a-5p has a negative regulation of MMP-14 expression.

To determine whether miR-181a-5p targets the MMP-14 3′-UTR directly, a luciferase reporter gene construct was generated by fusing the 3′-UTR to the firefly luciferase cDNA. miR-181a-5p significantly reduced luciferase activity in cells expressing the 3′-UTR containing reporter gene. However, this effect was not observed when the 7 nucleotide miR-181a-5p response element within the 3′UTR was converted to the complimentary sequence, confirming the direct relation of miR-181a-5p to MMP-14′s 3′UTR.

Functionally, MMP-14 enhances cell migration and activates MMP-2 which in turn promotes invasion and metastasis [6]. When miR-181a-5p reduces MMP-14, there is a corresponding reduction of active and intermediate MMP-2, along with an increased latent MMP-2. This is not due to increased expression of MMP-14's inhibitor, tissue inhibitor of metalloproteinase-2 (TIMP-2), as shown by Western blotting. As expected, miR-181a-5p expression reduced migration by transwell chamber assay, invasion by three-dimensional (3D)-invasion assay in highly invasive MDA-MB-231 breast cancer cells and HT-1080 human fibrosarcoma cells, and corresponded with decreased MMP-14 expression at the cell surface. The reduced migration and invasion potential of MDA-MB-231 and HT-1080 cells was also observed in vivo by use of the chick chorioallantoic membrane (CAM) invasion assay. Cancerous cells stably expressing miR-181a-5p failed to cross the chorionic epithelium and allantoic membrane, and also demonstrated impaired blood vessel formation.

To evidence the role of MMP-14 in human cancer dissemination, expression patterns in human cancer specimens was examined by immunohistochemistry (IHC). We observed that MMP-14 is minimally expressed in normal colonic mucosal cells. MMP-14 expresses at a low level in primary colon cancer cells, whereas expression dramatically increases in colon cancer cells located at the invasive front, or those that have already invaded into the submucosa. These observations provided evidence for the first time that MMP-14 contributes to cancer invasion in disease dissemination. The correlation of MMP-14 expression with miR-181a-5p in colon cancer progression is under investigation.

Utilizing this newly discovered interaction, miR-181a-5p may serve as a prognostic biomarker, or could possibly be mimicked by anti-metastatic therapeutics. The widespread function of miRNAs has been a target of several other studies in the pursuit of novel cancer treatments. Currently, there are multiple miRNA based replacement and inhibition therapeutics in the preclinical phase, along with Miravirsen, a phase II antiviral that targets miR-122 [7].

Several studies report mixed indications of miR-181 as a tumor suppressor [8] or promoter [5] depending on tumor type. This controversy may be the result of the complexity of miRNA regulation which can be altered by cellular context or synergistic affects. Additionally, most of these studies analysed bulk tissue which consists of normal epithelia and stromal cells, which we observed to lack the expression profile of cancerous cells, especially the invasive front.

The current discrepancy in function of miR-181a-5p is a problem that needs to be addressed. However, there are technical challenges considering the more than 500 PicTar predicted mRNA targets, and microarray studies showing less than 2 fold changes in miRNA expression is most common. This necessitates individual mRNA target characterization in order to validate the relation of cellular phenotype with altered miRNA expression. Utilizing this approach, our data demonstrates the negative modulation miR-181a-5p exerts on MMP-14 and consequential inhibition of metastatic traits including migration, invasion, and angiogenesis.
